# Correction to *Lancet Glob Health* 2017; 5: e593–603

**DOI:** 10.1016/S2214-109X(17)30228-0

**Published:** 2017-05-26

*Muhammad AH, Soofi S, Cousens S, et al. Community engagement and integrated health and polio immunisation campaigns in conflict-affected areas of Pakistan: a cluster randomised controlled trial.* Lancet Glob Health *2017*; **5:** e593–603—In this Article, the open access licence should have been listed as CC BY. This correction has been made to the online version as of May 26, 2017.

